# Cultural adaptation and preliminary psychometric assessment of the IMEVID questionnaire for assessing lifestyle in adults with type 2 diabetes in Peru

**DOI:** 10.1186/s41687-026-01162-6

**Published:** 2026-07-22

**Authors:** Eduardo José Carcausto Huamaní, Angie Yajaira Nieto Olivera, Nelson Fidel Diaz Reyes

**Affiliations:** 1 Faculty of Medicine, Universidad de San Martín de Porres, Lima, Peru; 2 Risk Management, Sanitas Peru, Lima, Peru; 3 Faculty of Medicine, Peruvian Union University, Lima, Peru; 4 Department of Medicine, Clinica Good Hope, Lima, Peru

**Keywords:** Type 2 diabetes, Patient-reported outcome measure, Self-reported outcome, Psychometrics, Score interpretation, Multidomain measurement, Lifestyle behavior, Peru

## Abstract

**Background:**

Multidomain patient-reported and self-reported instruments are often summarized using total scores, even when their domains represent conceptually distinct aspects of health behavior, emotional functioning, or self-management. Such scoring practices may obscure clinically meaningful domain-specific information if the instrument has not been shown to behave as a unidimensional reflective scale. This study culturally adapted the IMEVID questionnaire for use in Peru and evaluated its psychometric properties and score interpretation in adults with type 2 diabetes.

**Methods:**

We conducted a cross-sectional methodological study in 180 adults with self-reported type 2 diabetes in Peru. IMEVID underwent cross-cultural adaptation and expert-based content validity assessment. Psychometric evaluation included expert agreement for content validity, item and domain descriptive statistics, floor and ceiling effects, internal consistency estimates, Kaiser-Meyer-Olkin measures and Bartlett’s tests based on Pearson and polychoric correlation matrices, exploratory factor analysis, confirmatory factor analysis, and hypothesis-driven Spearman correlations among domains. Cronbach’s alpha was used for domains with three or more items, while inter-item Spearman correlations were used for two-item domains.

**Results:**

The adapted IMEVID showed high expert-based content validity agreement across clarity, coherence, relevance, and sufficiency criteria, with an overall mean agreement of 0.96 and acceptable overall internal consistency (α = 0.82). Domain-level internal consistency varied across the instrument, with Cronbach’s alpha ranging from 0.43 to 0.78 and two-item domain correlations ranging from 0.26 to 0.74. Factorability assessment differed according to the correlation matrix: the Pearson matrix showed acceptable sampling adequacy (KMO = 0.757), whereas the polychoric matrix yielded a very low KMO value (KMO = 0.14), likely influenced by sparse response patterns and marked ceiling effects in selected domains. Exploratory factor analysis was more consistent with a seven-factor solution aligned with the original IMEVID domains. Confirmatory factor analysis showed poor fit for the one-factor model (CFI = 0.677; TLI = 0.648; RMSEA = 0.104; SRMR = 0.151) and substantially better fit for the seven-domain model (CFI = 0.946; TLI = 0.936; RMSEA = 0.044; SRMR = 0.093). All standardized factor loadings in the seven-domain model were statistically significant.

**Conclusions:**

The Peruvian adaptation of IMEVID showed high content validity agreement, acceptable overall reliability, and preliminary structural support for the original seven-domain organization. The findings were more consistent with a multidomain lifestyle assessment tool than with a strictly unidimensional reflective scale. Domain scores may provide complementary information about distinct aspects of lifestyle and self-management. However, the structural findings should be considered preliminary, particularly given the very low polychoric KMO and the moderate sample size. Further validation is needed in larger clinically characterized samples.

**Supplementary Information:**

The online version contains supplementary material available at https://doi.org/10.1186/s41687-026-01162-6.

## Background

Patient-reported outcome measures (PROMs) are increasingly used to assess health-related experiences, behaviors, symptoms, and functioning from the patient perspective. Their usefulness, however, depends not only on feasibility or internal consistency, but also on whether the construct of interest is clearly defined and whether the resulting scores are interpreted in a psychometrically appropriate manner. Contemporary PROM methodology emphasizes that instrument development and validation should begin with an explicit theoretical framework and that any proposed domain or total score should be justified conceptually and empirically [1, 2, 17].

In type 2 diabetes (T2D), lifestyle-related behaviors remain central to disease management and long-term outcomes [3–6]. Eating patterns, physical activity, treatment-related behaviors, and emotional functioning all influence self-management, metabolic control, and the risk of complications [4–6]. Assessing lifestyle-related behaviors is complex because health-related choices may reflect both deliberate reasoning and automatic or context-dependent decision-making processes [18]. Several PROMs are available to assess diabetes-related behaviors, distress, or adherence, but many were originally developed in English-language settings and focus mainly on selected aspects of lifestyle rather than on a broader integrated profile. As summarized in Table 1, commonly used instruments such as the Summary of Diabetes Self-Care Activities (SDSCA), Diabetes Self-Management Questionnaire (DSMQ), Self-Care Inventory-Revised (SCI-R) primarily assess behavioral or treatment-related dimensions, whereas the Diabetes Distress Scale (DDS) and Problem Areas in Diabetes (PAID) focus predominantly on diabetes-related emotional burden [7–11]. In contrast, IMEVID was originally conceived in Spanish as a multidomain instrument integrating both behavioral and emotional components relevant to lifestyle in adults with T2D [12].

**Table 1:** Conceptual overview of selected diabetes-related behavioral, emotional, and lifestyle instruments

Instrument	Year introduced	Country/language of origin	Main construct	Primary measurement orientation	Includes emotional dimension	Includes behavioral dimension	Spanish availability	Broad international use
SDSCA	2000	USA / English	Diabetes self-care activities (recent behaviors)	Behavioral self-care profile	No	Yes	Yes	Very high
DDS	2005	USA / English	Diabetes distress	Distress measure	Yes	No	Yes	Very high
PAID	1995	USA / English	Diabetes-related emotional distress	Distress measure	Yes	No	Yes	High
DSMQ	2013	Germany / German	Diabetes self-management	Multidomain self-management measure	Limited	Yes	Yes (translations available)	Moderate to high
SCI-R	2002	USA / English	Diabetes self-care / regimen adherence	Self-care inventory	No	Yes	Partial / limited	Moderate
IMEVID	2003	Mexico / Spanish	Lifestyle in adults with T2D	Lifestyle-related multidomain behavioral profile	Yes	Yes	Yes	Primarily Latin American use

Abbreviations: SDSCA, Summary of Diabetes Self-Care Activities; DDS, Diabetes Distress Scale; PAID, Problem Areas in Diabetes; DSMQ, Diabetes Self-Management Questionnaire; SCI-R, Self-Care Inventory-Revised; IMEVID, Instrumento para Medir el Estilo de Vida en Diabéticos

Note. This table is intended to provide conceptual context for the measurement problem addressed in this study. It is not a systematic review or a formal comparative assessment of measurement properties

This distinction highlights a fundamental measurement issue: when instruments combine conceptually heterogeneous domains, their total scores may not necessarily reflect a single latent construct but may instead summarize related yet non-interchangeable behavioral components. This issue is particularly relevant in lifestyle measurement, where behaviors, emotional states, and knowledge may coexist within the same person without necessarily reflecting a single underlying trait [14–16].

The IMEVID (*Instrumento para Medir el Estilo de Vida en Diabéticos*) was originally developed in Mexico as a self-administered instrument specifically designed for adults with T2D. The original development study reduced the questionnaire to 25 items grouped into seven domains: nutrition, physical activity, tobacco use, alcohol consumption, diabetes-related information, emotions, and treatment adherence. The authors reported expert-based content evaluation, internal consistency, and test-retest reliability, and proposed the instrument as a practical tool for lifestyle assessment in diabetes [12]. Subsequent work supported its construct validity by showing associations between higher total scores and more favorable anthropometric and metabolic parameters, including body mass index, waist-to-hip ratio, glycated hemoglobin, fasting glucose, cholesterol, and triglycerides [13].

After its initial development, IMEVID was also used in descriptive studies, educational interventions, and even in populations beyond its original target group, such as kidney transplant recipients [25–27]. This applied use suggests that the instrument has practical value. At the same time, its broader uptake appears to have outpaced a critical re-examination of its measurement structure and score interpretation under contemporary psychometric standards. In particular, it remains unclear whether the total score should be understood as representing a coherent unidimensional lifestyle construct or whether IMEVID is more appropriately interpreted as a multidomain behavioral profile.

In Peru, no formal cultural adaptation and psychometric re-examination of IMEVID has been reported. Given the linguistic commonality but contextual differences between Mexico and Peru, adaptation requires more than literal equivalence; it also requires confirmation that item wording, content, and score interpretation remain appropriate in the new setting. More broadly, re-evaluating IMEVID in Peru offers an opportunity not only to adapt the instrument culturally, but also to clarify how its scores should be interpreted.

This study therefore uses IMEVID as an applied methodological case to examine a broader measurement problem: how multidomain lifestyle instruments should be interpreted when their domains are clinically related but not psychometrically interchangeable manifestations of a single latent variable.

The aim of this study was to culturally adapt the IMEVID questionnaire for use in Peru and to provide a preliminary assessment of its content validity agreement, reliability, structural validity, and score interpretation in adults with type 2 diabetes. Particular attention was given to whether the adapted instrument supported a strictly unidimensional interpretation or whether its scores were more appropriately interpreted as a multidomain lifestyle profile aligned with the original seven-domain organization.

## Methods

### Study design

We conducted a methodological study to culturally adapt IMEVID for use in Peru and to re-examine its measurement properties in adults with type 2 diabetes (T2D). The study was carried out in two sequential phases: (1) cultural adaptation and content evaluation, and (2) psychometric evaluation. The study was designed and reported in accordance with the COSMIN framework for studies on measurement properties of patient-reported outcome measures (PROMs) [1,2].

### Study population and data collection

Participants were recruited using a nonprobabilistic convenience sampling strategy through three main channels: invitations in outpatient clinics to patients attending physicians and endocrinologists, dissemination through social media groups of people with diabetes, and open online dissemination. The survey was administered online, and participation was voluntary and anonymous. Eligible participants were adults aged 18 years or older residing in Peru who reported a previous medical diagnosis of type 2 diabetes. Because recruitment was conducted partly through clinical settings and partly through open online channels, the sample may include participants with different levels of healthcare access, digital literacy, and motivation for diabetes self-care. When necessary, participants were allowed to complete the questionnaire with caregiver assistance for practical support or clarification during survey completion. No formal sample size calculation was performed because the study was designed as a methodological assessment of measurement properties and score interpretation rather than as a hypothesis-driven comparative study. The final sample included 180 participants with complete responses to all 25 IMEVID items, corresponding to approximately seven participants per item. This sample size was considered adequate for descriptive analyses, internal consistency estimates, and hypothesis-driven interdomain correlations, but moderate for factor analytic modelling; therefore, EFA and CFA findings should be interpreted as preliminary.

No financial incentives were offered. The study followed the principles of the Declaration of Helsinki.

### Ethical considerations

The study protocol was approved by an institutional Research Ethics Committee under approval number XXX, dated XXX. Electronic informed consent was obtained from all participants before survey initiation.

The study protocol was approved by the Research Ethics Committee of the Faculty of Health Sciences, Universidad Peruana Unión, under approval number 2021-CE-FCS-UPeU-00184, dated April 5, 2021. Electronic informed consent was obtained from all participants before survey initiation.

### Instrument

IMEVID was originally developed in Mexico as a self-administered instrument to assess lifestyle in adults with T2D. Its self-administered format was intended to facilitate the rapid collection of clinically relevant information on lifestyle-related behaviors and emotional factors, while minimizing the need for consultation time and additional healthcare resources in outpatient care.

The original version consists of 25 items grouped into seven domains: nutrition, physical activity, tobacco use, alcohol consumption, diabetes-related information, emotions, and treatment adherence. Items are scored using a 3-point ordinal response format (0, 2, and 4), with higher scores reflecting more favorable responses. Domain scores are obtained by summing the items within each domain, and a total score can be calculated by summing all 25 items.

Because IMEVID includes conceptually distinct domains, the present study did not assume a priori that the total score necessarily represented a single reflective latent construct. Instead, the psychometric evaluation was explicitly oriented toward clarifying the most appropriate interpretation of the instrument’s scores in the Peruvian context.

### Phase 1: Cultural adaptation and content validity assessment

Before the adaptation process, the research team obtained authorization from the original developer of the IMEVID questionnaire to adapt the instrument for use in the Peruvian context. Because the original IMEVID was developed in Spanish, the process did not require translation into another language. Instead, it focused on cross-cultural adaptation, with emphasis on semantic, idiomatic, and contextual equivalence.

First, the original Spanish version was reviewed by two Spanish-speaking linguistic reviewers to identify words, expressions, food-related references, and formulations that could be interpreted differently in Peru. Their observations were synthesized and used to prepare a culturally adapted version. This preliminary version was then evaluated by a multidisciplinary expert panel.

Content validity was assessed through structured expert judgment. Four expert judges evaluated each item according to clarity, coherence, relevance, and sufficiency using dichotomous response options (Yes/No). Items with disagreement were reviewed qualitatively, and wording adjustments were incorporated during the cultural adaptation process.

### Phase 2: Psychometric evaluation

In the second phase, the adapted version of IMEVID was evaluated psychometrically in the study sample. Analyses focused on descriptive performance, internal consistency, factorability, exploratory and confirmatory structural validity, and hypothesis-testing for construct validity.

### Descriptive analysis

Descriptive statistics were calculated for each domain, including mean, standard deviation, median, range, skewness, and kurtosis. Because domain scores were derived from ordinal items and reflected behaviorally heterogeneous content, these statistics were interpreted as descriptive indicators of score distribution rather than as evidence of latent structure.

### Internal consistency

Internal consistency was assessed for the total score and for domains containing three or more items using Cronbach’s alpha. For two-item domains, inter-item Spearman correlations were calculated instead of Cronbach’s alpha, as alpha is not considered an appropriate estimator for two-item scales.

Given the multidomain nature of the instrument, internal consistency coefficients were interpreted cautiously. In line with contemporary PROM methodology, alpha coefficients were not taken as evidence that the full instrument necessarily measured a single unidimensional construct.

### Hypothesis-testing for construct validity

Construct validity was further examined through hypothesis-testing, as recommended by the COSMIN framework. Based on behavioral theory and prior literature on diabetes-related self-management and emotional burden [4, 5, 19–25], three a priori hypotheses were specified:

1. Emotional domain scores would show moderate positive correlations with treatment-related behaviors and nutrition, reflecting the expected influence of emotional functioning on self-management.
2. Treatment-related behaviors and nutrition would show a stronger association with each other than with substance-use domains.
3. Conceptually distinct domains, such as diabetes-related information and tobacco use, would show weak or negligible correlations, supporting discriminant validity.

Interdomain associations were analyzed using Spearman rank correlations because of the ordinal response structure and the non-normal distribution expected for some domains. Hypotheses were considered supported when the correlations were in the expected direction and within the anticipated magnitude range.

### Statistical analysis

All analyses were performed using R version 4.3.3 (R Foundation for Statistical Computing, Vienna, Austria). Descriptive statistics were calculated for participant characteristics, item responses, domain scores, and total IMEVID scores. Item and domain distributions were inspected for skewness, kurtosis, floor effects, ceiling effects, and restricted variance.

Content validity was assessed through expert agreement. For each item and criterion, agreement was calculated as the proportion of expert judges rating the item as adequate using dichotomous Yes/No judgments. Agreement was summarized separately for clarity, coherence, relevance, and sufficiency, and an overall mean agreement was calculated across all items and criteria. Because the expert-rating format was dichotomous rather than ordinal, these results were interpreted as content validity agreement rather than as Aiken’s V.

Internal consistency was assessed using Cronbach’s alpha for the total score and for domains containing three or more items. For two-item domains, Spearman inter-item correlations were calculated instead of Cronbach’s alpha.

Factorability was examined using the Kaiser-Meyer-Olkin measure of sampling adequacy and Bartlett’s test of sphericity. Because IMEVID items use a three-category ordinal response format, both Pearson and polychoric correlation matrices were examined. The polychoric matrix was interpreted cautiously because ordinal items with sparse categories, restricted variance, or marked floor or ceiling effects may produce unstable correlation estimates.

Although polychoric correlations are generally preferred for ordinal items, the very low polychoric KMO, together with sparse response combinations, restricted item variance, and marked ceiling effects in selected items, raised concerns about the stability of the polychoric correlation matrix in this sample. Therefore, the Pearson correlation matrix was used for the exploratory factor analysis as a pragmatic observed-score approach. The resulting factor solution was interpreted as preliminary and exploratory, rather than as definitive evidence of the dimensional structure of IMEVID.

Exploratory factor analysis was conducted on the Pearson correlation matrix using minimum residual extraction and oblimin rotation. The number of factors was informed by parallel analysis, eigenvalues, scree plot inspection, model fit, and clinical interpretability. A five-factor solution was examined based on parallel analysis, and a seven-factor solution was examined because the original IMEVID instrument was organized into seven theoretical domains.

Confirmatory factor analysis was performed using the WLSMV estimator for ordinal items. Two theoretically relevant models were compared: a one-factor model including all 25 items as indicators of a single lifestyle factor, and a seven-domain model corresponding to the original IMEVID domains. Model fit was evaluated using chi-square, degrees of freedom, comparative fit index, Tucker-Lewis index, root mean square error of approximation, and standardized root mean square residual. Standardized factor loadings were reported with standard errors, p-values, and confidence intervals.

Construct validity was further examined through hypothesis-testing using Spearman correlations among domain scores. Correlations were interpreted according to their direction, magnitude, and consistency with prespecified hypotheses. P-values were reported as exact values when applicable, and statistical significance was set at a two-sided alpha level of 0.05.

During manuscript preparation, an AI-assisted language model was used to support language editing and editorial refinement. The authors reviewed, verified, and approved all content and remain fully responsible for the manuscript.

## Results

### Participants

A total of 180 adults with self-reported type 2 diabetes completed all 25 IMEVID items and were included in the psychometric analyses. Participant characteristics are presented in Table 2. Twenty-four participants completed the questionnaire with assistance from a family member or caregiver, mainly for practical support or clarification during survey completion. Some non-IMEVID descriptive variables included missing or non-specified responses, particularly residence; these variables were reported descriptively and did not affect the psychometric analyses.

**Table 2:** Characteristics of the study sample (*N* = 180)

Characteristic	Category	*n* (%)
Sex	Women	90 (50.0)
	Men	90 (50.0)
Age, y	Mean (SD)	52.2 (17.9)
	Range	18–99
City of residence	Lima	73 (40.6)
	Other cities	51 (28.3)
	Not specified	56 (31.1)
Employment status	Employed	94 (52.2)
	Homemaker	41 (22.8)
	Retired	23 (12.8)
	Unemployed	18 (10.0)
	Not specified	4 (2.2)
Marital status	Married	81 (45.0)
	Single	56 (31.1)
	Cohabiting	15 (8.3)
	Widowed	14 (7.8)
	Divorced	10 (5.6)
	Not specified	4 (2.2)
Time since diagnosis	≤ 5 years	82 (45.6)
	6–10 years	47 (26.1)
	> 10 years	46 (25.6)
	Not specified	5 (2.8)
Questionnaire completed by	Participant	156 (86.7)
	Family member/caregiver	24 (13.3)
Current treatment*	Diet	64 (35.6)
	Metformin	75 (41.7)
	Insulin	41 (22.8)
	Other	50 (27.8)
	Liraglutide	4 (2.2)

*Treatment categories were not mutually exclusive; participants could report more than one treatment modality. Percentages were calculated using the full sample as denominator unless otherwise indicated

### Cultural adaptation and content validity agreement

Minor cultural and semantic adaptations were required to improve comprehensibility in the Peruvian context. These changes primarily involved wording adjustments and contextual substitutions to enhance clarity without altering the intended meaning of the original items.

The expert panel showed high content validity agreement across the adapted IMEVID items. Mean agreement was 0.95 for clarity, 0.99 for coherence, 0.97 for relevance, and 0.93 for sufficiency, with an overall mean agreement of 0.96 across all items and criteria. Item-level agreement values are provided in Supplementary Table S1.

Items with lower agreement for clarity, particularly those below 0.90, and items with qualitative comments suggesting semantic ambiguity or cultural mismatch were reviewed by the research team. Wording adjustments were incorporated when needed to improve comprehension while preserving the intended meaning of the original items. The lowest agreement was observed for item 4, mainly because of cultural differences in the relevance of the original tortilla-related wording in the Peruvian context. The main semantic and cultural adaptations are summarized in Supplementary Table S2.

### Descriptive performance and floor/ceiling effects

Descriptive statistics for the IMEVID domains and floor/ceiling effects are presented in Table 3. Score distributions showed variability across domains. The tobacco domain showed marked negative skewness and a strong ceiling effect, with 161 participants (89.4%) achieving the maximum score. Ceiling effects were also observed in alcohol consumption (30.6%) and treatment-related behaviors (27.8%). These distributional patterns were considered relevant for interpreting the factorability analyses, particularly the discrepancy between Pearson and polychoric correlation matrices. Item-level descriptive statistics and floor/ceiling effects are presented in Supplementary Table S3.

**Table 3:** Descriptive Statistics for IMEVID domains and floor/ceiling effects

Domain	Mean	SD	Median	Range	Skewness	Kurtosis	Floor (%)	Ceiling (%)
Nutrition	23.67	6.16	24	4–36	-0.53	0.42	0.0	1.7
Physical activity	6.31	3.21	6	0–12	-0.04	-0.77	5.0	8.3
Tobacco	7.62	1.26	8	0–8	-3.92	16.59	1.1	89.4
Alcohol	4.83	2.46	4	0–8	-0.01	-1.11	5.0	30.6
Diabetes-related information	3.44	2.25	4	0–8	0.42	-0.45	12.8	9.4
Emotional status	6.93	3.34	8	0–12	-0.43	-0.57	7.2	10.6
Treatment-related behaviors	11.44	4.20	12	0–16	-0.70	-0.37	1.7	27.8

Note. Floor and ceiling effects represent the percentage of participants with the minimum and maximum possible domain scores, respectively

### Internal consistency

The overall internal consistency of the IMEVID total score was acceptable (Cronbach’s alpha = 0.82). Domain-level reliability estimates are presented in Table 4. For domains with three or more items, Cronbach’s alpha coefficients ranged from 0.43 to 0.78. The physical activity domain showed the lowest internal consistency (alpha = 0.43), whereas treatment-related behaviors and emotional status showed higher internal consistency estimates.

**Table 4:** Internal consistency estimates for IMEVID domains and total score

Domain	Items (codes)	No. of items	Statistic	Coefficient
Nutrition	p01–p09	9	Cronbach α	0.677
Physical activity	p10–p12	3	Cronbach α	0.433
Tobacco	p13–p14	2	Inter-item Spearman correlation	0.538
Alcohol	p15–p16	2	Inter-item Spearman correlation	0.743
Diabetes-related information	p17–p18	2	Inter-item Spearman correlation	0.261
Emotional status	p19–p21	3	Cronbach α	0.731
Treatment-related behaviors	p22–p25	4	Cronbach α	0.784
Total IMEVID score	p01–p25	25	Cronbach α	0.820

Note. Cronbach α was reported only for domains with 3 or more items. For 2-item domains (tobacco, alcohol, and diabetes-related information), inter-item Spearman correlations were reported because they are more appropriate than Cronbach α for 2-item scales

For two-item domains, Cronbach’s alpha was not used. Instead, inter-item Spearman correlations were calculated. Correlations were moderate for tobacco (rho = 0.54) and strong for alcohol (rho = 0.74). By contrast, the diabetes-related information domain showed a weaker inter-item association (rho = 0.26), suggesting greater conceptual independence between the two knowledge-related items.

### Structural validity

Structural validity results are summarized in Table 5. Factorability assessment yielded different results depending on the correlation matrix used. Based on the observed-score Pearson correlation matrix, the Kaiser-Meyer-Olkin measure indicated acceptable sampling adequacy (KMO = 0.757), and Bartlett’s test of sphericity was statistically significant, χ²(300) = 1272.79, *p* < 0.001. In contrast, the polychoric correlation matrix yielded a very low KMO value (KMO = 0.14), despite a statistically significant Bartlett’s test, χ²(300) = 9722.70, *p* < 0.001. This discrepancy may partly reflect the three-category ordinal response format of IMEVID and sparse or highly asymmetric response distributions in selected items. However, the very low polychoric KMO remains an important limitation and indicates that the structural analyses should be interpreted cautiously. The complete item-level correlation matrix is provided in Supplementary Table S4.

**Table 5:** Structural validity: factorability, exploratory factor analysis, and confirmatory factor analysis

Analysis	Matrix / model	χ²	df	*p*-value	KMO	RMSR or SRMR	CFI	TLI	RMSEA	RMSEA 95% CI
Factorability	Pearson correlation matrix	1272.79	300	< 0.001	0.757	—	—	—	—	—
Factorability	Polychoric correlation matrix	9722.70	300	< 0.001	0.140	—	—	—	—	—
Exploratory factor analysis	Five-factor model	—	—	—	—	0.043	—	0.890	0.043	0.028–0.057
Exploratory factor analysis	Seven-factor model	—	—	—	—	0.034	—	0.943	0.031	0.000–0.049
Confirmatory factor analysis	One-factor model	808.88	275	< 0.001	—	0.151	0.677	0.648	0.104	0.096–0.112
Confirmatory factor analysis	Seven-domain model	343.63	254	< 0.001	—	0.093	0.946	0.936	0.044	0.032–0.056

Abbreviations: CFA, confirmatory factor analysis; CFI, comparative fit index; EFA, exploratory factor analysis; KMO, Kaiser-Meyer-Olkin measure of sampling adequacy; RMSEA, root mean square error of approximation; RMSR, root mean square of the residuals; SRMR, standardized root mean square residual; TLI, Tucker-Lewis index

Note. RMSR is reported for exploratory factor analysis models, whereas SRMR is reported for confirmatory factor analysis models. The Pearson matrix was used for EFA because it showed acceptable sampling adequacy, while the polychoric matrix was interpreted cautiously because of sparse response patterns and marked ceiling effects in selected domains

Exploratory factor analysis was conducted using the Pearson correlation matrix. Eigenvalues suggested several factors above unity, although the eighth eigenvalue was marginal. Therefore, factor retention was guided by eigenvalues, parallel analysis, model fit, and interpretability rather than by the Kaiser criterion alone. A five-factor solution showed acceptable fit indices (RMSR = 0.043; TLI = 0.890; RMSEA = 0.043; 95% CI: 0.028–0.057). A seven-factor solution, consistent with the original IMEVID domain structure, showed better fit (RMSR = 0.034; TLI = 0.943; RMSEA = 0.031; 95% CI: 0.000–0.049). The seven-factor solution was also more clinically interpretable because it broadly reflected the original multidomain organization of IMEVID. Given the very low polychoric KMO and the use of the Pearson correlation matrix for EFA, these findings should be regarded as preliminary and hypothesis-generating rather than confirmatory. Full EFA loadings for the five-factor and seven-factor solutions are provided in Supplementary Tables S5 and S6.

Confirmatory factor analysis was conducted to compare a one-factor model with the original seven-domain IMEVID structure. The one-factor model showed poor fit, χ² = 808.88, df = 275, *p* < 0.001; CFI = 0.677; TLI = 0.648; RMSEA = 0.104; SRMR = 0.151. In contrast, the seven-domain model showed substantially better fit, χ² = 343.63, df = 254, *p* < 0.001; CFI = 0.946; TLI = 0.936; RMSEA = 0.044; SRMR = 0.093.

Standardized factor loadings in the seven-domain model were statistically significant for all items (Table 6). Loadings ranged from 0.364 to 0.688 for nutrition, from 0.330 to 0.745 for physical activity, from 0.891 to 0.935 for tobacco, from 0.913 to 0.928 for alcohol, from 0.625 to 0.626 for diabetes-related information, from 0.744 to 0.868 for emotional status, and from 0.624 to 0.878 for treatment-related behaviors. The lowest standardized loading was observed for item p12 in the physical activity domain. One-factor CFA loadings are provided in Supplementary Table S7.

**Table 6:** Standardized factor loadings for the seven-domain confirmatory factor analysis model

Domain	Item	Standardized loading	SE	z-value	p-value	95% CI
Nutrition	p01	0.595	0.067	8.918	<0.001	0.464–0.725
Nutrition	p02	0.461	0.068	6.804	<0.001	0.328–0.593
Nutrition	p03	0.376	0.079	4.751	<0.001	0.221–0.532
Nutrition	p04	0.592	0.06	9.834	<0.001	0.474–0.710
Nutrition	p05	0.688	0.048	14.379	<0.001	0.595–0.782
Nutrition	p06	0.364	0.083	4.396	<0.001	0.202–0.527
Nutrition	p07	0.542	0.062	8.792	<0.001	0.421–0.663
Nutrition	p08	0.524	0.071	7.364	<0.001	0.384–0.663
Nutrition	p09	0.428	0.08	5.338	<0.001	0.271–0.586
Physical activity	p10	0.745	0.128	5.835	<0.001	0.495–0.995
Physical activity	p11	0.417	0.096	4.335	<0.001	0.229–0.606
Physical activity	p12	0.33	0.099	3.343	0.001	0.137–0.524
Tobacco	p13	0.891	0.085	10.482	<0.001	0.725–1.058
Tobacco	p14	0.935	0.111	8.39	<0.001	0.717–1.153
Alcohol	p15	0.913	0.067	13.685	<0.001	0.782–1.044
Alcohol	p16	0.928	0.065	14.24	<0.001	0.800–1.056
Diabetes-related information	p17	0.625	0.147	4.244	<0.001	0.337–0.914
Diabetes-related information	p18	0.626	0.136	4.594	<0.001	0.359–0.893
Emotional status	p19	0.744	0.069	10.826	<0.001	0.609–0.878
Emotional status	p20	0.805	0.055	14.71	<0.001	0.698–0.912
Emotional status	p21	0.868	0.069	12.637	<0.001	0.733–1.003
Treatment-related behaviors	p22	0.83	0.034	24.619	<0.001	0.764–0.896
Treatment-related behaviors	p23	0.856	0.039	21.721	<0.001	0.779–0.933
Treatment-related behaviors	p24	0.624	0.068	9.226	<0.001	0.492–0.757
Treatment-related behaviors	p25	0.878	0.035	25.354	<0.001	0.810–0.945

Abbreviations: CFA, confirmatory factor analysis; CI, confidence interval; SE, standard error

Note: The CFA model was estimated using the WLSMV estimator for ordinal items. All standardized factor loadings were statistically significant. Confidence intervals exceeding 1.00 in selected domains should be interpreted cautiously in the context of ordinal estimation, sparse response patterns, and marked ceiling effects

Overall, the EFA and CFA findings were more consistent with the original seven-domain organization than with a strictly unidimensional model. However, these structural findings should be considered preliminary.

### Hypothesis-testing for construct validity

Interdomain correlations were examined to test the prespecified hypotheses (Table 7). As hypothesized, the emotional status domain showed moderate positive correlations with treatment-related behaviors and nutrition, supporting the expected relationship between emotional functioning and self-management-related behaviors.

**Table 7:** Interdomain Spearman correlations with 95% confidence intervals and p-values

Domain	Nutrition	Physical activity	Tobacco	Alcohol	Diabetes-related information	Emotional status	Treatment-related behaviors
Nutrition	1	0.217 (0.073 to 0.352); *p* = 0.003	0.218 (0.074 to 0.353); *p* = 0.003	0.275 (0.134 to 0.405); *p* = 0.0002	0.059 (-0.088 to 0.204); *p* = 0.430	0.331 (0.194 to 0.455); *p* = 6.0 × 10^-6	0.663 (0.572 to 0.738); *p* = 3.88 × 10^-24
Physical activity	—	1	-0.114 (-0.256 to 0.033); *p* = 0.128	-0.053 (-0.198 to 0.094); *p* = 0.477	0.295 (0.156 to 0.423); *p* = 5.7 × 10^-5	0.248 (0.106 to 0.381); *p* = 0.0008	0.273 (0.132 to 0.403); *p* = 0.0002
Tobacco	—	—	1	0.128 (-0.018 to 0.270); *p* = 0.086	-0.069 (-0.213 to 0.078); *p* = 0.360	0.016 (-0.131 to 0.162); *p* = 0.832	0.264 (0.123 to 0.395); *p* = 0.0003
Alcohol	—	—	—	1	0.016 (-0.131 to 0.162); *p* = 0.833	0.025 (-0.122 to 0.171); *p* = 0.738	0.296 (0.157 to 0.424); *p* = 5.3 × 10^-5
Diabetes-related information	—	—	—	—	1	0.141 (-0.005 to 0.282); *p* = 0.058	0.110 (-0.037 to 0.252); *p* = 0.143
Emotional status	—	—	—	—	—	1	0.306 (0.167 to 0.433); *p* = 2.9 × 10^-5
Treatment-related behaviors	—	—	—	—	—	—	1

Abbreviations: CI, confidence interval

Note: Values are Spearman rho coefficients with approximate 95% confidence intervals and p-values. Correlations were calculated using domain scores. The upper triangular matrix is shown to avoid duplicate values. P-values are reported as exact values when computationally available

Consistent with the second hypothesis, the strongest observed association was between treatment-related behaviors and nutrition, exceeding the correlations observed between these domains and substance-use domains. This pattern was clinically plausible and supported the expected clustering of self-management-related behavioral domains.

In accordance with the third hypothesis, conceptually distinct domains showed weak or negligible associations. For example, diabetes-related information showed limited association with several other domains, and its correlation with emotional status was small. This pattern supported discriminant validity while also suggesting partial behavioral alignment between some domains, such as diabetes-related information and physical activity.

Overall, the direction and magnitude of the interdomain correlations were consistent with most prespecified hypotheses. Rather than supporting a single homogeneous construct, this pattern was more compatible with a multidomain instrument in which domains remain meaningfully related while retaining conceptual distinctiveness.

## Discussion

This study culturally adapted the IMEVID questionnaire for use in Peru and evaluated its psychometric properties and score interpretation in adults with type 2 diabetes. The findings should be interpreted in continuity with the original Mexican development study, which proposed IMEVID as a practical self-administered instrument for lifestyle assessment in adults with type 2 diabetes.

Overall, the adapted IMEVID showed a coherent but nuanced psychometric pattern. Content validity agreement was high across clarity, coherence, relevance, and sufficiency. The total score showed acceptable internal consistency, and most prespecified hypotheses for construct validity were supported. Structural analyses were more consistent with the original seven-domain organization than with a strictly unidimensional interpretation. EFA results were compatible with a seven-factor solution, and CFA showed better fit for the seven-domain model than for the one-factor model. These findings provide preliminary support for interpreting the adapted IMEVID as a multidomain lifestyle assessment tool rather than as a purely unidimensional reflective scale.

A central methodological finding was the discrepancy between the KMO values obtained from the Pearson and polychoric correlation matrices. Although the polychoric matrix yielded a very low KMO value, the Pearson matrix showed acceptable sampling adequacy and was used as a pragmatic observed-score basis for exploratory factor analysis. This discrepancy may partly reflect the three-category ordinal response format of IMEVID and to sparse or highly asymmetric response distributions in selected items. The tobacco domain showed a particularly marked ceiling effect, and ceiling effects were also observed in alcohol consumption and treatment-related behaviors. These distributional features, together with restricted item variance and sparse bivariate response patterns, may affect the stability of polychoric correlation estimates and, consequently, the KMO derived from them. Problematic item coding cannot be fully excluded as a potential contributor; however, item directionality was reviewed during the scoring and adaptation process, and the observed pattern appears more consistent with distributional constraints than with systematic coding error. Therefore, the low polychoric KMO should be interpreted as a warning regarding sparse ordinal correlation patterns and the limitations of factorability assessment under these conditions, rather than as conclusive evidence that structural exploration of the instrument is inappropriate.

The factor-analytic results were more consistent with a multidomain interpretation of IMEVID than with a strictly unidimensional model. The one-factor confirmatory model showed poor fit, indicating that the 25 items should not be interpreted as interchangeable indicators of a single homogeneous latent lifestyle construct. In contrast, the original seven-domain model showed substantially better fit, with acceptable CFI, TLI, and RMSEA values. All standardized factor loadings in the seven-domain model were statistically significant. These findings are consistent with the original conceptual organization of IMEVID into nutrition, physical activity, tobacco use, alcohol consumption, diabetes-related information, emotional status, and treatment-related behaviors. However, these results should still be interpreted as preliminary because some domains contain only two items, the sample size was moderate, and selected domains showed marked ceiling effects.

The lower internal consistency observed in selected domains should be interpreted in light of item content rather than as a simple measurement failure. In the physical activity domain, for example, one item refers more directly to exercise frequency, whereas other items capture broader aspects of daily occupation, mobility, time spent outside the home, or sedentary behavior. These behaviors are clinically related but may not function as interchangeable indicators of a single narrow construct. Similarly, the diabetes-related information domain combines attendance at formal educational sessions with the patient’s perceived effort to obtain diabetes-related information. These two indicators may be related but not equivalent, because attendance at structured education depends partly on access to educational opportunities, whereas information-seeking may occur through medical consultations, reading, informal advice, or independent searching.

This domain-oriented interpretation may provide actionable information for individualized counseling and intervention planning, particularly in outpatient diabetes care. One of the strengths of IMEVID is its self-administered format, which allows the collection of behavioral and emotional information without substantially increasing consultation time or requiring additional healthcare personnel. In settings where structured lifestyle assessment is limited by time, resources, or access barriers, a brief multidomain instrument may help clinicians identify the areas that require more focused counseling, referral, or follow-up.

The cultural adaptation process also contributes to the literature. Although the original and adapted versions share the Spanish language, linguistic commonality does not guarantee semantic, idiomatic, or contextual equivalence. Food-related references, everyday expressions, and examples of health behaviors may differ across Spanish-speaking countries. The initial linguistic review and subsequent expert evaluation allowed the adaptation to prioritize conceptual equivalence over literal preservation of wording. The high expert agreement observed across clarity, coherence, relevance, and sufficiency supports the appropriateness of the adapted items for the Peruvian context.

This study has several strengths. It provides the first cultural adaptation and preliminary psychometric assessment of IMEVID in Peru. The analysis combined content validity agreement, item and domain descriptive statistics, floor and ceiling effects, internal consistency estimates, factorability assessment using both Pearson and polychoric matrices, exploratory factor analysis, confirmatory factor analysis, and hypothesis-driven interdomain correlations. This integrated approach allowed a more complete evaluation of score interpretation than would have been possible using internal consistency alone.

Several limitations should also be acknowledged. First, the cross-sectional design does not allow evaluation of responsiveness, predictive validity, or temporal stability in the Peruvian sample. Although test-retest reliability was assessed in the original Mexican validation study, the temporal stability of the culturally adapted Peruvian version remains to be established. Second, the sample was nonprobabilistic and based on self-reported type 2 diabetes, which may limit generalizability and may not fully represent all adults with clinically confirmed type 2 diabetes receiving care in Peru. Third, recruitment through outpatient clinic invitations, social media diabetes groups, and open online dissemination may have introduced selection bias. Participants with greater healthcare contact, internet access, digital literacy, or interest in diabetes self-care may have been more likely to participate.

Fourth, objective clinical markers such as HbA1c were not available in this dataset, which prevented examination of known-groups validity or criterion-related associations in the Peruvian sample. Fifth, a minority of participants completed the questionnaire with caregiver assistance, which may have influenced how some items were interpreted. Sixth, although EFA and CFA findings were more consistent with the original seven-domain organization, the structural analysis should be considered preliminary because of the moderate sample size, the ordinal three-category item format, sparse response patterns in selected items, and the presence of domains defined by only two indicators, as established in the original IMEVID design. This does not imply that the instrument should be modified; rather, it indicates that latent variables represented by only two items are generally less stable, more dependent on model constraints, and more difficult to evaluate reliably than factors represented by three or more indicators. Therefore, the factor loadings and structural interpretation of these domains should be viewed with caution. Finally, the expert content evaluation used dichotomous judgments; therefore, content validity was summarized as expert agreement rather than through ordinal rating-based coefficients.

Future studies should build on these findings in several ways. First, they should examine test-retest reliability and responsiveness in Peruvian samples to determine whether the adapted instrument is stable over time and sensitive to change after intervention. Second, studies including metabolic and clinical outcomes would help clarify the external validity and practical usefulness of domain scores and total scores in relation to glycated hemoglobin, lipid parameters, emergency visits, hospitalizations, and other clinically relevant outcomes. Third, larger clinically characterized samples should be used to confirm the seven-domain structure and to examine whether any higher-order summary score is psychometrically defensible. Finally, prospective studies may help determine whether changes in total or domain-level scores are informative for monitoring risk and therapeutic response over time.

## Conclusions

The adapted IMEVID showed high content validity agreement, acceptable overall internal consistency, and preliminary structural support for the original seven-domain organization in a Peruvian sample of adults with self-reported type 2 diabetes. EFA and CFA findings were more consistent with a multidomain interpretation than with a strictly unidimensional reflective model.

These findings do not invalidate the use of a total score as a pragmatic summary of lifestyle-related behaviors. However, they suggest that domain-level interpretation may provide more clinically actionable information for individualized assessment and intervention, particularly when identifying specific areas such as nutrition, physical activity, emotional well-being, diabetes-related information, or treatment adherence.

Further validation work is needed in larger clinically characterized samples to confirm the seven-domain structure and to determine how total and domain-level scores can best inform diabetes care.

## Supplementary Information

Below is the link to the electronic supplementary material.

Supplementary Material 1

Supplementary Material 2

Supplementary Material 3

Supplementary Material 4

Supplementary Material 5

Supplementary Material 6

Supplementary Material 7

Supplementary Material 8

Supplementary Material 9

Supplementary Material 10

## Data Availability

No datasets were generated or analysed during the current study.

## Abbreviations

DDS: Diabetes distress scale
DSMQ: Diabetes self-management questionnaire
IMEVID: Instrumento para medir el estilo de vida en diabéticos
KMO: Kaiser-meyer-olkin
PAID: Problem areas in diabetes
PROM: Patient-reported outcome measure
SCI-R: Self-care inventory-revised
SDSCA: Summary of diabetes self-care activities
T2D: Type 2 diabetes
