# Supplementary material for: Cultural adaptation and preliminary psychometric assessment of the IMEVID questionnaire for assessing lifestyle in adults with type 2 diabetes in Peru
Source: J Patient Rep Outcomes. 2026 Jul 22;10:177. doi: 10.1186/s41687-026-01162-6 (PMC13631132; doi:10.1186/s41687-026-01162-6)
Supplement: Supplementary file 1 [file 41687_2026_1162_MOESM5_ESM.docx]

**Supplementary Table S2. Key semantic and cultural adaptations of the IMEVID questionnaire for the Peruvian context.**

| **Item** | **Original domain** | **Modification** | **Rationale** |  | **Expert agreement / qualitative note** |
| --- | --- | --- | --- | --- | --- |
| p04 | Nutrition | “tortillas” changed to “handfuls of rice and/or potatoes” | Replacement of a culturally specific food item with a locally meaningful equivalent |  | This item showed the lowest agreement and was reviewed qualitatively because the original food reference was not fully equivalent in the Peruvian context. |
| p10 | Physical activity | Added a minimum daily exercise time (30 minutes) | Clarification of the expected minimum duration of daily physical activity |  | Expert review supported the need for clearer wording regarding the minimum duration of physical activity. |
| p16 | Alcohol | “alcoholic beverages” changed to “glasses of alcoholic beverages” | Improved quantification of alcohol intake |  | Expert review supported the wording adjustment to improve response interpretation. |
| p17 | Diabetes-related information | Lexical adjustment | Improved semantic clarity |  | Expert review supported minor lexical refinement to improve clarity without changing item meaning. |

**Note.** Content validity was assessed by four expert judges using dichotomous Yes/No judgments across clarity, coherence, relevance, and sufficiency. Results were summarized as expert agreement rather than as Aiken’s V. All 25 items were evaluated. Items with lower agreement for clarity or qualitative comments suggesting semantic ambiguity or cultural mismatch were reviewed qualitatively, and wording adjustments were incorporated during the cultural adaptation process.
