# Supplementary material for: Cultural adaptation and preliminary psychometric assessment of the IMEVID questionnaire for assessing lifestyle in adults with type 2 diabetes in Peru
Source: J Patient Rep Outcomes. 2026 Jul 22;10:177. doi: 10.1186/s41687-026-01162-6 (PMC13631132; doi:10.1186/s41687-026-01162-6)
Supplement: Supplementary file 2 [file 41687_2026_1162_MOESM6_ESM.docx]

**Supplementary Table S3. Item-level descriptive statistics and floor/ceiling effects for the adapted IMEVID questionnaire**

| **Domain** | **Item** | **Mean** | **SD** | **Median** | **Observed range** | **Skewness** | **Kurtosis** | **Floor n (%)** | **Ceiling n (%)** |
| --- | --- | --- | --- | --- | --- | --- | --- | --- | --- |
| Nutrition | p01 | 2.52 | 1.18 | 2 | 0–4 | -0.14 | -0.55 | 14 (7.8) | 61 (33.9) |
| Nutrition | p02 | 2.79 | 1.28 | 2 | 0–4 | -0.56 | -0.66 | 15 (8.3) | 86 (47.8) |
| Nutrition | p03 | 2.58 | 1.44 | 2 | 0–4 | -0.49 | -0.98 | 28 (15.6) | 80 (44.4) |
| Nutrition | p04 | 2.37 | 1.28 | 2 | 0–4 | -0.18 | -0.65 | 23 (12.8) | 56 (31.1) |
| Nutrition | p05 | 2.44 | 1.55 | 2 | 0–4 | -0.40 | -1.24 | 38 (21.1) | 78 (43.3) |
| Nutrition | p06 | 3.02 | 1.31 | 4 | 0–4 | -0.99 | -0.19 | 16 (8.9) | 108 (60.0) |
| Nutrition | p07 | 2.47 | 1.27 | 2 | 0–4 | -0.23 | -0.68 | 20 (11.1) | 62 (34.4) |
| Nutrition | p08 | 2.20 | 1.23 | 2 | 0–4 | -0.06 | -0.44 | 26 (14.4) | 44 (24.4) |
| Nutrition | p09 | 3.28 | 1.05 | 4 | 0–4 | -1.03 | -0.07 | 4 (2.2) | 119 (66.1) |
| Physical activity | p10 | 1.91 | 1.59 | 2 | 0–4 | 0.08 | -1.43 | 61 (33.9) | 53 (29.4) |
| Physical activity | p11 | 2.49 | 1.44 | 2 | 0–4 | -0.40 | -1.03 | 30 (16.7) | 74 (41.1) |
| Physical activity | p12 | 1.91 | 1.65 | 2 | 0–4 | 0.08 | -1.53 | 65 (36.1) | 57 (31.7) |
| Tobacco | p13 | 3.78 | 0.73 | 4 | 0–4 | -3.42 | 11.78 | 3 (1.7) | 163 (90.6) |
| Tobacco | p14 | 3.84 | 0.72 | 4 | 0–4 | -4.68 | 21.13 | 5 (2.8) | 171 (95.0) |
| Alcohol | p15 | 2.50 | 1.17 | 2 | 0–4 | -0.11 | -0.51 | 14 (7.8) | 59 (32.8) |
| Alcohol | p16 | 2.33 | 1.49 | 2 | 0–4 | -0.27 | -1.17 | 37 (20.6) | 67 (37.2) |
| Diabetes-related information | p17 | 1.02 | 1.39 | 0 | 0–4 | 0.99 | -0.33 | 109 (60.6) | 21 (11.7) |
| Diabetes-related information | p18 | 2.42 | 1.40 | 2 | 0–4 | -0.31 | -0.96 | 29 (16.1) | 67 (37.2) |
| Emotional status | p19 | 1.93 | 1.43 | 2 | 0–4 | 0.05 | -1.06 | 49 (27.2) | 43 (23.9) |
| Emotional status | p20 | 2.30 | 1.29 | 2 | 0–4 | -0.15 | -0.68 | 26 (14.4) | 53 (29.4) |
| Emotional status | p21 | 2.70 | 1.41 | 2 | 0–4 | -0.60 | -0.83 | 24 (13.3) | 87 (48.3) |
| Treatment-related behaviors | p22 | 2.99 | 1.35 | 4 | 0–4 | -0.97 | -0.28 | 18 (10.0) | 107 (59.4) |
| Treatment-related behaviors | p23 | 2.18 | 1.51 | 2 | 0–4 | -0.15 | -1.25 | 44 (24.4) | 60 (33.3) |
| Treatment-related behaviors | p24 | 3.17 | 1.27 | 4 | 0–4 | -1.23 | 0.35 | 14 (7.8) | 119 (66.1) |
| Treatment-related behaviors | p25 | 3.11 | 1.25 | 4 | 0–4 | -1.09 | 0.07 | 13 (7.2) | 113 (62.8) |

**Note.** IMEVID items are scored from 0 to 4. Floor effect was defined as the proportion of participants scoring 0 on each item, and ceiling effect as the proportion scoring 4. Values are based on complete responses from 180 participants.
